# Stationary Atrial Fibrillation Properties in the Goat Do Not Entail Stable or Recurrent Conduction Patterns

**DOI:** 10.3389/fphys.2018.00947

**Published:** 2018-07-27

**Authors:** Arne van Hunnik, Stef Zeemering, Piotr Podziemski, Jorik Simons, Giulia Gatta, Laura Hannink, Bart Maesen, Marion Kuiper, Sander Verheule, Ulrich Schotten

**Affiliations:** Department of Physiology, Cardiovascular Research Institute Maastricht, Maastricht University, Maastricht, Netherlands

**Keywords:** atrial fibrillation, mapping, conduction patterns, stationary patterns, recurrence quantification analysis, AF dynamics

## Abstract

**Introduction:** Electro-anatomical mapping of the atria is used to identify the substrate of atrial fibrillation (AF). Targeting this substrate by ablation in addition to pulmonary vein ablation did not consistently improve outcome in clinical trials. Generally, the assessment of the substrate is based on short recordings (≤10 s, often even shorter). Thus, targeting the AF substrate assumes spatiotemporal stationarity but little is known about the variability of electrophysiological properties of AF over time.

**Methods:** Atrial fibrillation (AF) was maintained for 3–4 weeks after pericardial electrode implantation in 12 goats. Within a single AF episode 10 consecutive minutes were mapped on the left atrial free wall using a 249-electrode array (2.25 mm inter-electrode spacing). AF cycle length, fractionation index (FI), lateral dissociation, conduction velocity, breakthroughs, and preferentiality of conduction (Pref) were assessed per electrode and AF property maps were constructed. The Pearson correlation coefficient (PCC) between the 10 AF-property maps was calculated to quantify the degree spatiotemporal stationarity of AF properties. Furthermore, the number of waves and presence of re-entrant circuits were analyzed in the first 60-s file. Comparing conduction patterns over time identified recurrent patterns of AF with the use of recurrence plots.

**Results:** The averages of AF property maps were highly stable throughout the ten 60-s-recordings. Spatiotemporal stationarity was high for all 6 property maps, PCC ranged from 0.66 ± 0.11 for Pref to 0.98 ± 0.01 for FI. High stationarity was lost when AF was interrupted for about 1 h. However, the time delay between the recorded files within one episode did not affect PCC. Yet, multiple waves (7.7 ± 2.3) were present simultaneously within the recording area and during 9.2 ± 11% of the analyzed period a re-entrant circuit was observed. Recurrent patterns occurred rarely and were observed in only 3 out of 12 goats.

**Conclusions:** During non-self-terminating AF in the goat, AF properties were stationary. Since this could not be attributed to stable recurrent conduction patterns during AF, it is suggested that AF properties are determined by anatomical and structural properties of the atria even when the conduction patterns are very variable.

## Introduction

Mapping of conduction patterns has been of fundamental importance to understand mechanisms that maintain cardiac arrhythmias (Nattel et al., 2005). The importance of cardiac mapping in clinical practice was demonstrated by Haïssaguerre's et al. (1998) pivotal finding that atrial fibrillation (AF) was often initiated from the pulmonary veins (PV) and that ablation of ectopic sites in the PVs terminated AF. However, many patients experience recurrences of AF in the months following a PV isolation (Verma et al., 2015), suggesting that other regions in the atria may contribute to AF perpetuation as well.

Detailed mapping studies of AF, in both animal models (Berenfeld et al., 2000; Verheule et al., 2010) and humans (Konings et al., 1994; Allessie et al., 2010; Lee et al., 2014, 2015), have described fast, irregular and seemingly random conduction patterns. AF maintenance can be explained by different conceptual models such as multiple wavelets (Allessie et al., 1996, 2010), rotor activity (Jalife, 2011) and repetitive focal activity (Lee et al., 2015). Notably, these mechanisms are not mutually exclusive and different mechanisms may occur in an individual patient. Several electrophysiological parameters have been used to detect local sources of AF. Nedios et al. (2016) Complex fractionated atrial electrograms (CFAE) and high frequency zones are thought to reflect driver sites of rotational or focal activity or to correlate with areas demonstrating high complexity of AF. Unlike such electrogram parameters, with focal impulse and rotor mapping (FIRM) conduction patterns are identified that putatively describe focal and rotational activity. Narayan et al. (2012); Swarup et al. (2014) Unfortunately, targeting these substrate parameters has variable outcomes in clinical trials and need further validation (Gadenz et al., 2017; Krummen et al., 2017; van der Does and de Groot, 2017).

A potential factor contributing to the limited success rates are the limitations of the mapping techniques used. The atrial surface can only be mapped with a limited time and spatial resolution. A limited spatial resolution may lead to misinterpretation of conduction patterns (Kuklik et al., 2017; Roney et al., 2017). If higher spatial resolution is obtained, for example by point-by-point mapping typically relatively short recordings are acquired. It is unknown whether these recordings are representative for longer episodes of AF. Also, it is largely unexplored whether AF driver sites are stable across different AF episodes.

In this study we analyzed left atrial epicardial high-density recording in goats with 3 to 4 weeks of maintained AF. We studied the degree of stationarity of local AF properties derived from ten 60-s-long recordings within a single AF episode and between different AF episodes. Moreover, we analyzed complexity and stability of conduction patterns, and rotational activity. For the assessment of stability of conduction patterns, we made use of a new recurrence quantification analysis.

## Materials and methods

### Animal model

This study was carried out in accordance with the principles of the Basel Declaration and regulations of European directive 2010/63/EU. The local ethical board for animal experimentation of the Maastricht University approved the protocol. In total 12 goats, 6 per study, weighing 60 ± 9.8 kg were included. Goats were anesthetized (sufentanyl 6 μg/kg/h and propofol 5–10 mg/kg/h, i.v.) and electrodes were implanted on the pericardium above the left atrium (LA). After 2 weeks of recovery from surgery, AF was induced by repetitive burst of stimuli (1 s, 50 Hz, 2 times threshold with a maximum of 10 V) using subcutaneously implanted neurostimulator (Itrel 3 or 4, Medtronic, Minneapolis, Minnesota, USA). AF was subsequently maintained for 3–4 weeks. For open chest sacrifice experiments, goats were anesthetized with parenteral sufentanyl 6 μg/kg/h, propofol 10 mg/kg/h, and rocuronium 0.3 mg/kg/h.

### Data acquisition

**Figure 1:**
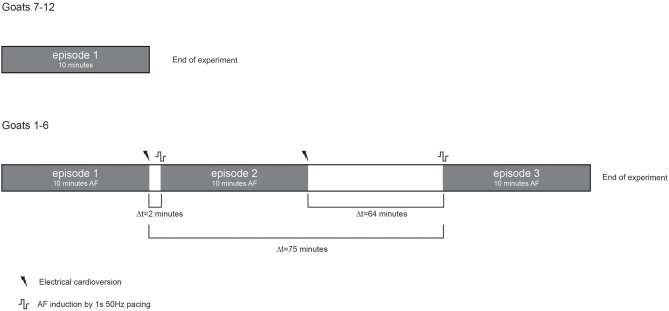
Schematic presentation of the experimental protocol. Grey boxes represent periods of AF. Ten consecutive minutes per AF episode were recorded. Only a single AF episode was recorded in goats with ID numbers 7–12, see also Table 2. Three different AF episodes were recorded in goats with ID number 1–6. Two episodes were close in time and 1 episode was separated for about 1 h.

A circular mapping array containing 249 electrodes (2.4 mm inter-electrode distance, 14.3 cm^2^ surface area) was placed on the left atrial (LA) free wall and kept in stable position throughout the experiment. Unipolar electrograms were recorded with 1.039 kHz sampling rate, bandwidth of 0.1–408 Hz and AD resolution of 16bit. In a 10-min window ten 60-s files were recorded during non-selfterminating AF. In a subset of 6 animals 2 additional 10-min windows were recorded (Figure 1). Electrical cardioversion of AF was performed using a ≤20Joules synchronized DC shock (Physio-control lifepak 9 B, Medtronic, Minneapolis, Minnesota, USA). The DC shock was delivered on endocardial catheters of which one was placed in the coronary sinus and the other in the right atrial cavity.

### Analysis of local activation time and direction

The recorded signals were analyzed offline using custom-made analysis software (MATLAB 8.1, The Mathworks, Inc., Natick, Massachusetts, USA). Local deflections, activation times, and unipolar fractionation index (FI) were identified using a probabilistic annotation algorithm, as previously described. Zeemering et al. (2012); Lau et al. (2015) Based on the activation times, the AF cycle length (AFCL) and conduction direction and velocity (CV) were determined. For calculation of conduction vectors, a plane was fitted through the central activation time and its direct neighbors in space and time (min. 3 max 8). If the conduction time of a neighboring electrode implied a CV of <20 cm/s, the occurrence of conduction block was assumed and the activation time of that neighbor was excluded from plane fitting. Per electrode the degree for preferential direction of conduction (Pref) was calculated as Pref = 1-(circular variance of all conduction vectors). The maximal time difference of activation times with its neighbors was calculated as a measure of epicardial lateral dissociation (LD).

### AF property maps analysis

Based on the results of the above-mentioned analysis, 6 different parameters, i.e., FI, AFCL, LD, CV, breakthroughs (BT), and Pref were obtained for each individual electrode and used to construct AF property maps. Per parameter the spatial Pearson correlation coefficient (PCC) between property maps, of all 45 possible comparisons within a 10-min window, was determined and averaged per animal, producing an intra-episode correlation of property maps at various time points. To address potential correlations due to chance, we randomly reassigned electrodes in space and recalculated the average PCC. In a subset of 6 animals three 10-min windows were recorded from 3 different episodes of AF, in order to determine the inter-episode correlation.

### Analysis of epicardial waves and re-entrant circuits

Next, we analyzed the fibrillation waves propagating on the epicardial surface as previously described by Zeemering et al. (2012) In short, waves were defined as clusters of activation times that are connected in space and time by an apparent CV of > 20cm/s. The earliest activation time that cannot be explained by its surrounding was identified as the starting point of the wave. This starting point was specified as peripheral, on the border of the electrode, or breakthrough, separated >1 electrode from the border, (BT) origin. Furthermore, we analyzed re-entrant activity based on conduction paths that can be identified based on the activation times. Conduction paths were determined as the shortest contiguous trajectory between a starting and end point of a wave, considering CV ≥ 20 cm/s. If the trajectory had ≥1 intersection(s) it was considered as a re-entrant circuit (RC).

### Recurrence analysis

**Figure 2:**
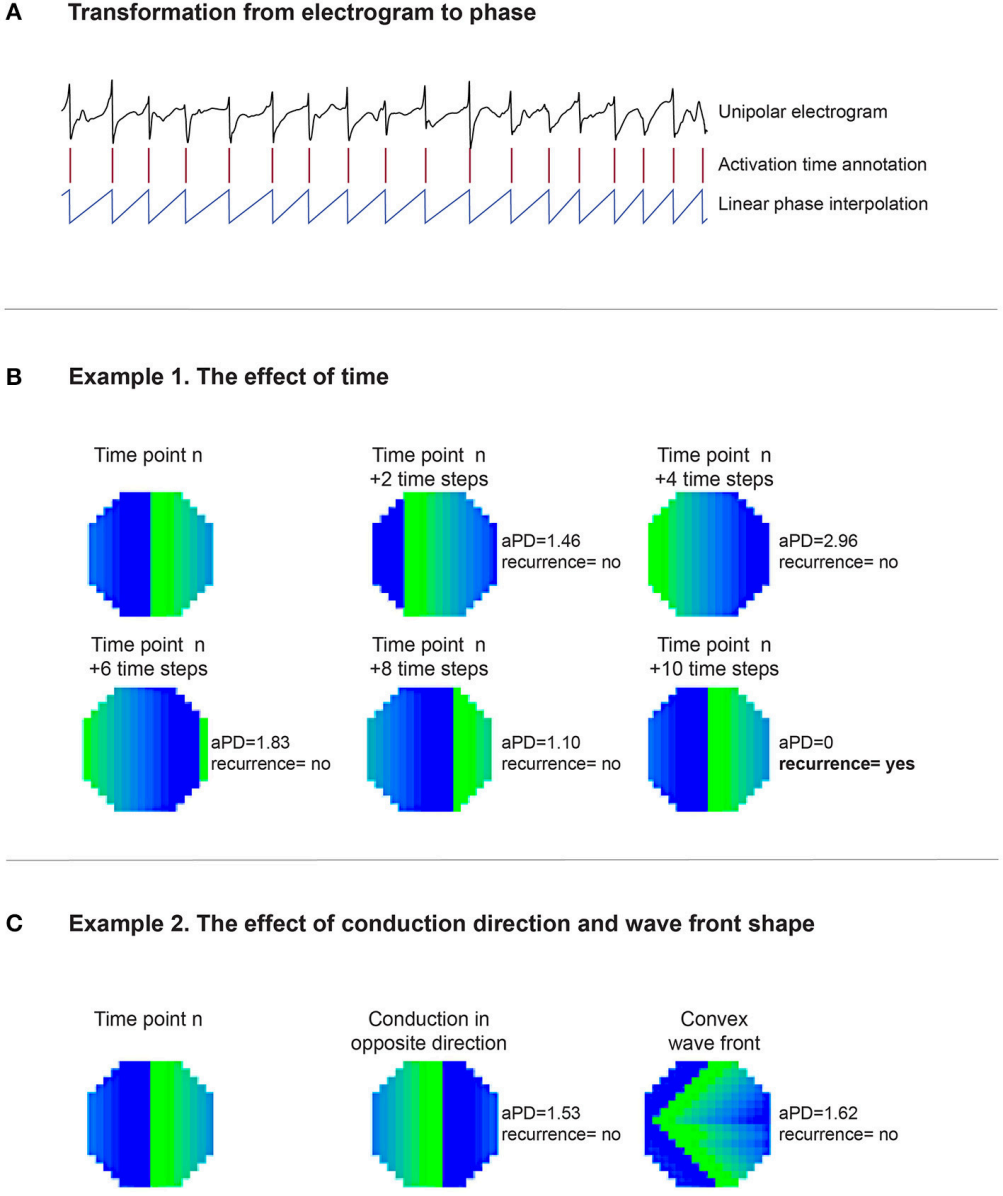
**(A)** Illustrates that based on the unipolar electrogram activation times (red columns) are identified. Based on the length of the interval, the linear phase from activation time to activation time was interpolated. **(B)** Demonstrates the effect of time on the recurrence analysis. One large “recurrent” wave front was present in the middle mapping area in the first map, timepoint *n*. In the second map, *n* + *2 timesteps*, the wave front propagated somewhat further, resulting in an aPD of 1.46. This level of the aPD is above π/4 (0.7854) and therefore considered to be non-recurrent. At timestep *n* + *4*, the aPD increased to a maximum of 2.96. In the following timesteps (*n* + *6 and n* + *8)*, aPD decreased again until at *n* + *10* the wave front reoccurred at the same site like timepoint *n*. Here, the aPD was 0 and consequently considered to be recurrent. **(C)** Demonstrates the effect of direction and wave front shape. Map *n* is presented again as the first map. The wave front in the second map propagated in the opposite direction compared to map *n*. The aPD was now calculated to be 1.53, thus not recurrent. In the third map the wave did conduct in the same direction like map *n*. However, the wave front shape was now set to be convex instead of planer. The aPD between this map and map *n* was 1.62, thus also non-recurrent.

For a recording with N time samples, a recurrence plot (an N × N scatter plot with time on the x- and y-axis) can be constructed. If an event occurs at two time points with sufficient similarity, it is considered to be recurrent and can be identified by a dot in the scatter plot. Here, we analyzed the recurrence of conduction patterns in 60-s-recordings. We considered a pattern to be recurrent if the wave front(s); (1) reached the same point in space, (2) propagated in the same direction, and (3) had a similar shape. To achieve this, we developed a method that is based on local activation times and corresponding activation intervals. The linear phase (−π to π) was interpolated for every individual interval (Figure 2A). This approach does not require Hilbert transformation or time embedding of the raw electrogram as frequently used for the identification of phase singularities.

Phase signals were down-sampled to 10 timesteps per AFCL because the recurrence analysis leads to a large number of comparisons [(60s^*^1.039kHz)^2^ = 3.9e9] and long computation times would be required. Based on the phase signals, phase maps were constructed for all time steps. For individual electrodes, the difference in phase was calculated by comparing 2 phase maps. Based on all 249 phase differences, the average phase difference (aPD) was computed. This was executed for every possible comparison between time steps within the 60-s-recording. Recurrent patterns were identified at an aPD of < π/4, corresponding to 1/8 of the AFCL. The rate of recurrent patterns was calculated as the ratio of the number of observed recurrences to the number of expected recurrences if the pattern was fully recurrent. The number of unique recurrent patterns was subjectively scored (by AvH) with the help of conduction movies.

To illustrate the impact of conduction patterns on the aPD and consequent recurrence identification, we can consider the two hypothetical examples presented in Figures 2B,C.

**Example 1**, (Figure 2B) demonstrates the effect of time in the recurrence analysis. One large “recurrent” wave front was present in the middle mapping area in the first map, timepoint *n*. In the second map, *n* + *2 timesteps*, the wave front had propagated somewhat further, resulting in an aPD of 1.46. This level of the aPD is above π/4 (0.7854) and therefore considered to be non-recurrent. At timestep *n* + *4*, the aPD increased to a maximum of 2.96. In the following timesteps (*n* + *6 and n* + *8)*, aPD decreased until at *n* + *10* the wave front reoccurred at the same site like timepoint *n*. Here, the aPD was 0 and consequently the pattern was considered to be recurrent.

**Example 2** (Figure 2C) demonstrates the effect of direction and wave front shape. Map *n* is again presented as the first map. The wave front in the second map propagated in the opposite direction compared to map *n*. The aPD was now calculated to be 1.53, thus non-recurrent. In the third map the wave did propagate in the same direction like map *n*. However, the wave front shape was now set to be convex instead of planar. The aPD between this map and map *n* was 1.62, therefore also non-recurrent.

### Statistics

Data are presented as mean ± sd. Data were tested for normality using a Kolmogorov-Smirnov test. The effect of time was tested using a repeated measure ANOVA. Intra- and inter-episode correlation between property maps was assessed using Pearson correlation coefficient (PCC). A Bonferroni's correction was applied to correct for multiple comparisons. Inter-episode correlation differences were tested with a Wilcoxon rank sum test.

## Results

### AF property maps

**Figure 3:**
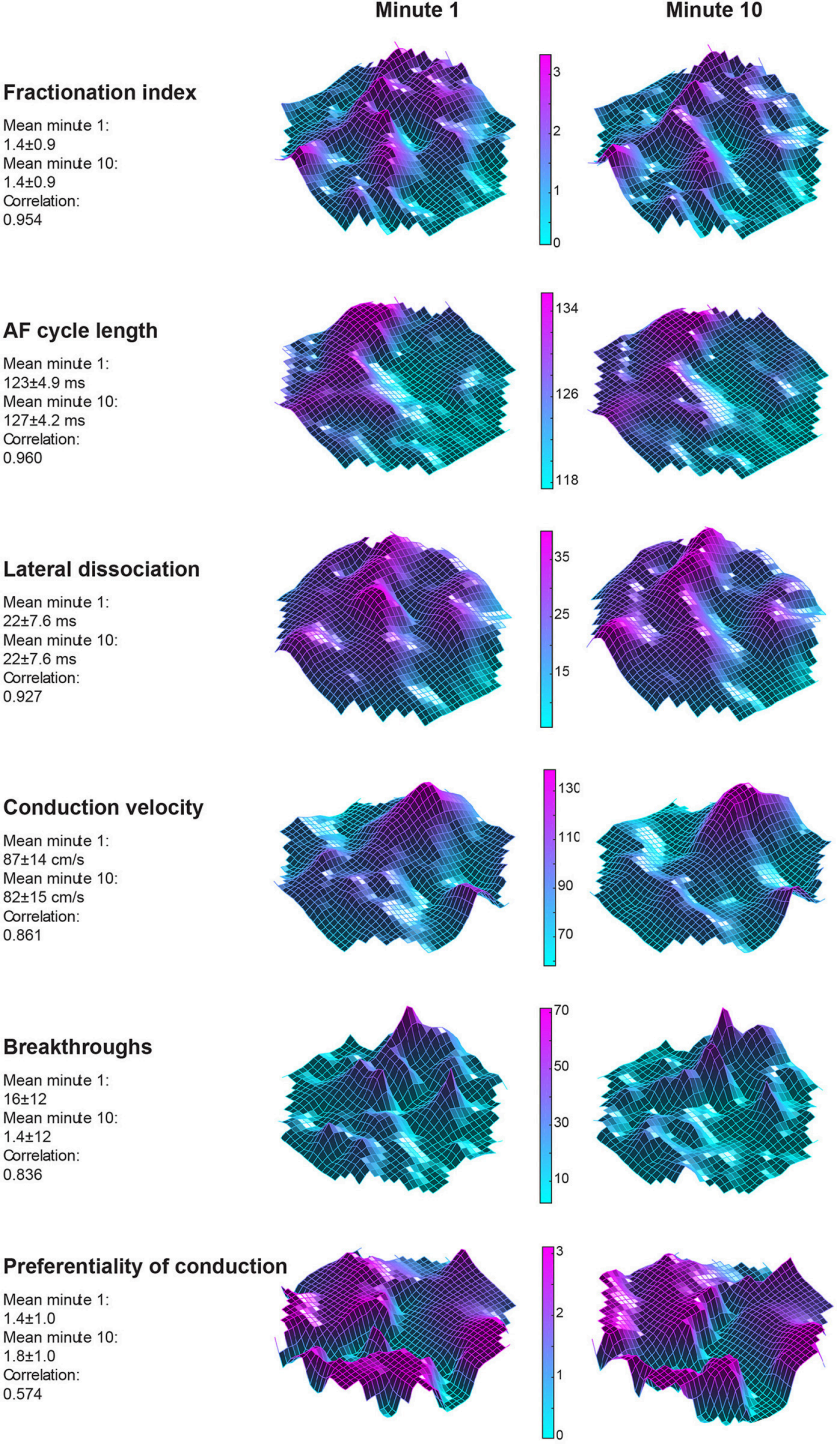
Spatial distribution of 6 AF parameters from goat 2. The left sided column represents property maps of the first 60-s-recording in a 10-min window. The right column depicts the final minute of the 10-min window. A high agreement for the absolute average of the maps was found between the maps. Interestingly, also a high degree of stationarity of spatial organization was found when the Pearson correlation coefficient (PCC) was considered. This was true for almost all parameters but weakest for preferentiality of conduction direction, suggesting highly variable conduction patterns.

**Figure 4:**
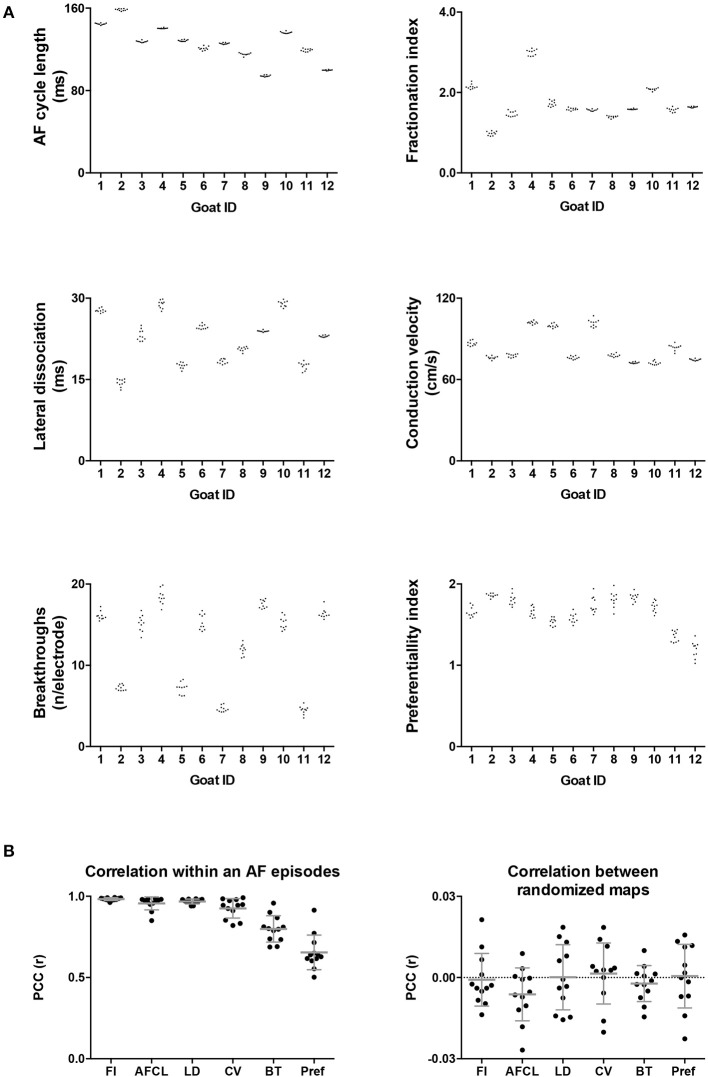
**(A)** Intra-episode PCC for 6 different AF parameters. On the x-axis the individual animals are given. Panel A represents the absolute average of a map in a 60-s-recording. Per animal 10 values of the 10 consecutive maps are given. **(B)** In the first panel the average PCC, based on 45 correlations, is given per animal per parameter. The right-hand panel confirms that this high degree of stationarity did not occur due to chance since breaking the spatial coherence completely removed the correlation.

Twelve goats a 10-min period within a single AF episode was recorded. This 10-min period was divided into ten 60-s-recordings. In Figure 3 two property maps, the first and last recording in a period, of goat 2 are presented. Only minor differences in the spatial distribution of the AF properties between the 2 recordings are apparent. This suggests that the AF properties remained very stable over this interval of 9 min. This high stability was found in all 6 parameters and all goats (Figure 4). We further addressed the spatial stationarity of the different parameters by exploring the correlation between AF property maps. From Figure 4B it can be appreciated that the average PCCs were very high, ranging from 0.66 ± 0.11 for Pref up to 0.98 ± 0.01 for FI and all PCCs were significant (Table 1). To underscore that this finding was not due to chance we broke the spatial coherence of the maps by randomly reassigning the electrodes within the map. This test diminished the average PCCs to almost 0 (right panel of Figure 4B). These observations demonstrate that both a high temporal and spatial stationarity was present.

**Table 1:** Spatial variation (coefficient of variation and range) and stability in time (PCC) of the 6 different AF parameters.

	**Coefficient of variation**	**Range**	**PCC(r)**	**PCC *p*-value**
Fractionation index	50 ± 0.8%	4.60 ± 1.5	0.983 ± 0.01	*p* < 0.001
AFCL (ms)	4.3 ± 2%	21.2 ± 8.2	0.956 ± 0.04	*p* < 0.001
Lateral dissociation (ms)	28 ± 8%	29.8 ± 5.5	0.968 ± 0.01	*p* < 0.001
Conduction velocity (cm/s)	23 ± 14%	115 ± 76	0.926 ± 0.06	*p* < 0.001
BT (n/electrode/60 s)	78 ± 34%	53 ± 22	0.799 ± 0.08	*p* < 0.001
Preferentiality	53 ± 11%	3.1 ± 0.02	0.655 ± 0.11	*p* < 0.001

*Per animal the average coefficient of variation and average range (max-min) of the 10 maps was calculated. The grand mean ± sd is presented in the table. The grand mean of the Pearson correlation coefficient (PCC) was based on all 45 comparisons per animal*.

We also looked at to what extent AF properties vary in space. High correlations between 60-s-recordings could have occurred due to a limited spatial diversity of the parameter. Table 1 presents the coefficient of variation and range within the property maps. Within the AFCL-maps the coefficient of variation was very low (4.3 ± 2%), demonstrating a fairly homogeneous spatial distribution. By contrast, for FI-, preferentiality-, and breakthrough-maps the coefficient of variation was much larger, ranging from 50 to 78%. This indicates that discrete zones on the atrial epicardial surface exhibited different properties. In Table 1 the overall characteristics of the 6 different property maps is presented.

All property maps are available in the Supplemental Materials. Raw data are not available online because of the large data size, 240 min for 249 unipolar channels at a sampling frequency of 1039 Hz. The raw data supporting the conclusions of this manuscript will be made available by the authors, without undue reservation, to any qualified researcher.

### Short term AF dynamics

**Figure 5:**
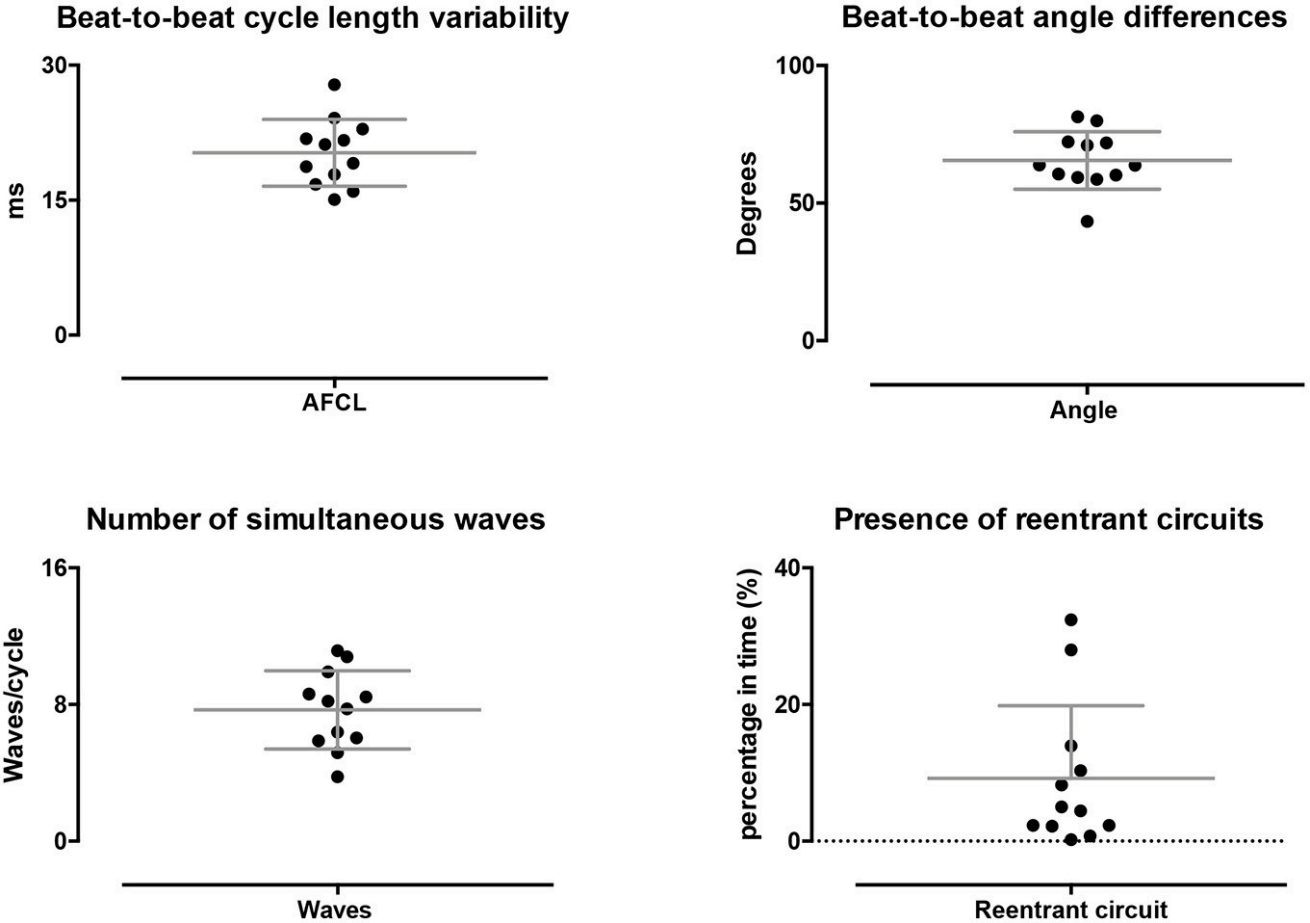
Dynamic properties of conduction during AF. On a beat-to-beat level spatiotemporal variability was found, as reflected by the beat-to-beat difference AFCL and direction of conduction. Furthermore, on average multiple waves were present at every beat. A limited percentage of time re-entrant circuits were present and showed a high degree of variation.

Potentially, stable conduction properties can be stable on a beat-to-beat level or at somewhat longer timespans, e.g., seconds, could have contributed to the stationary property maps within single AF episodes. Therefore, we explored dynamic behavior of AF (Figure 5). On a beat-to-beat level, the AFCL varied by 20 ± 3.7 ms, 16 ± 3.6% of the AFCL, and conduction direction varied with 66 ± 10 degrees. This high dynamic behavior was also reflected in the large average number of simultaneously present waves per cycle, 7.7 ± 2.3. In total 453 re-entrant trajectories were found of which 25 lasted >2 rotations. On average re-entrant activity was present for 9.2 ± 11%. The lifespan of re-entrant circuits was 149 ± 18 ms. Hence, the average lifespan of re-entries was short and close to the AFCL with a lifespan to AFCL ratio of 1.2 ± 0.3. These finding demonstrated a large beat-to-beat variability with high dynamic patterns that cannot explain stationary AF properties.

### Recurrence of conduction patterns

On average, AF exhibited a highly dynamic behavior in terms frequency and patterns, nonetheless a high degree of spatiotemporal stationarity of AF properties was present. This could be explained by frequent recurrent patterns that dominate the property maps. We therefore investigated the occurrence of recurrent AF patterns.

**Figure 6:**
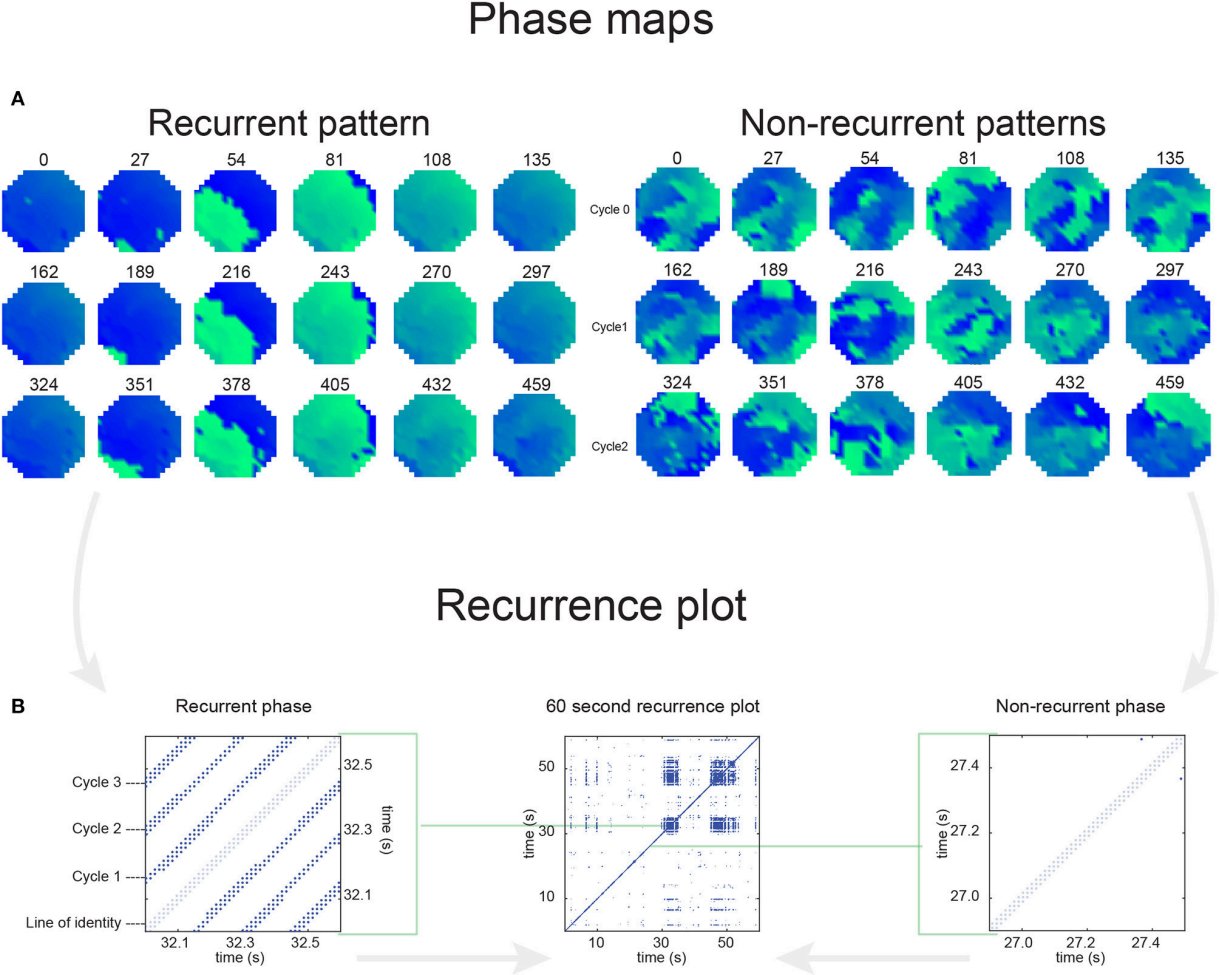
A schematic explanation of recurrence analysis of conduction patterns. Goat 10, Table 2, was used for this example. **(A)** Based on all 249 electrodes a phase angle map for each time instance, sample, was constructed. In the phase maps, clearly conducting wave fronts can be identified. The analysis was performed with time steps of AFCL/10 (13.5 ms). In the depicted phase map, we used AFCL/5 (27 ms) for clarity of the illustration. In the left-hand series of phase maps a period with recurrent activity is illustrated. Three consecutive waves start at the lower left-hand corner conducting toward the right-hand upper corner. This recurrent phase was present for 13 cycles. In the right-hand series of phase maps, a phase with non-recurrent activity is depicted. Multiple wave fronts were simultaneously present propagating in different directions. As can be appreciated from these maps no recurrent patterns were present. **(B)** Recurrence plots, a N × N scatter plot with time on the x and y-axis, were constructed by comparing each time instance with any other time instance. A recurrent pattern is visualized by a blue dot. The recurrence plot always has a central diagonal line, line of identity, because at this timepoint it is compared to itself and is by definition recurrent. Two timepoints were considered to be recurrent if the average phase angle difference (aPD) was less than π/4. **Recurrent phase**. Because of the periodic nature of conduction during a recurrent phase diagonal lines are formed in the plot. The maximal line length within this phase reflects the duration of the recurrent phase. The periodic behavior also leads to multiple diagonal lines, one for each cycle. Therefore, a recurrent phase will appear as a square around the line of identity. During the **non-recurrent phase** all time instances display unique patterns. In the recurrence plot this is reflected by the lack of recurrences or single dots (a random recurrence by chance), as can be seen in the right upper corner. Within the 60-s-recording, represented by the middle recurrence plot, a majority of the time no recurrence was found. However, at about 32, 48, and 52 s large recurrence blocks can be seen. These blocks exhibited the pattern as depicted in the left panel A. All recurrence blocks showed the same pattern.

In Figure 6 a representation of the construction of a representative recurrence plot is presented. A segment of the AF recording with recurrent activity is depicted on the left series of maps in panel A. In the right side a series of maps with a segment with non-recurrent activity is presented. In Figure 6B, the recurrence plots over 3 cycles of these two segments are shown. The recurrent phase presented diagonal lines while during the segment with chaotic activity no recurrences were found. In the overall 60s-recurrence plot, we can observe that the non-recurrent phase was present throughout most of the time. The recurrent pattern appeared 3 times, at timepoints 32, 48, and 52s, in the 60-s-recordings.

**Figure 7:**
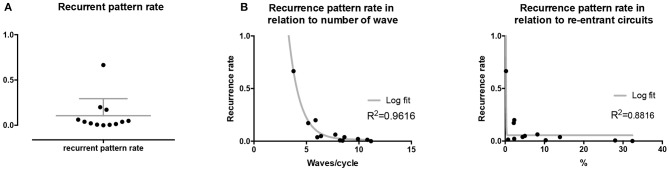
Recurrent pattern rates of conduction patterns. **(A)** Recurrent pattern rates were generally low with only 3 out of 9 animals expressing higher rates. **(B)** Recurrent pattern rates as such had a strong, logarithmic association with the number of waves, but not with re-entrant circuits.

Recurrence plots, wave and re-entrant circuit analysis for each individual animal are presented in Table 2. In the majority of goats (9 out of 12, goat number; 1, 3, 4, 6, and 8-12) no frequent or long lasting recurrent patterns were found. Only 3 goats had higher recurrence rates. Overall 1.6 ± 0.7 recurrent patterns per goat were found, with either peripheral or BT origins. Sixty-four percent of the reentrant circuits with >2 rotations were identified as recurrent events. No predominant origin of conduction pattern was found. Frequent disruption occurred, initiating unique states resulting in a recurrent pattern rate of 0.11 ± 0.19 (Figure 7). The association of recurrent pattern rate was strong with the number of waves but was weak with the number of reentrant circuits.

**Table 2:** Overview of recurrence, wave and re-entry analysis for all 12 goats.

**Goat ID**	**Recurrence map**	**Predominant recurrent pattern**	**Secondary recurrent pattern**	**Tertiary recurrent pattern**	**Rate of recurrent patterns**	**Waves/cycle**	**AFCL (ms)**	**RC presence (% in time)**	**RC lifespan (ms)**
**1**	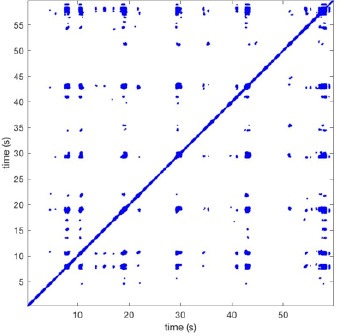	**Wave type:** PW **Origin:** NE → SW **Time point:** 58	NA	NA	0.021	9.9	150	2.30	159
**2**	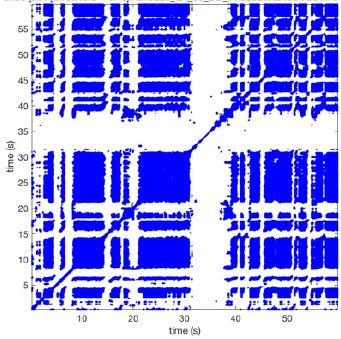	**Wave type:** rBT **Origin:** NW **Time point:** 4 10 21 etc.	**Wave type:** PW Origin: SW → NE **Time point:** 26	**Wave type:** PW **Origin:** E → W **Time point:** 55.9	0.665	3.8	164	0.21	137
**3**	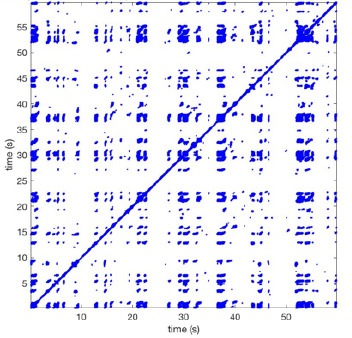	**Wave type:** rBT **Origin:** NW **Time point:** 1 21 51 etc.	**Wave type:** PW **Origin:** SE → NW **Time point:** 8.5	NA	0.063	7.8	132	8.21	176
**4**	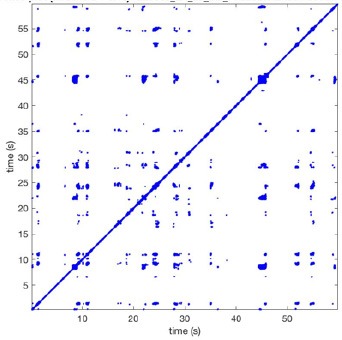	**Wave type:** PW **Origin:** NE → SW **Time point:** 45	**Wave type:** PW **Origin:** SW → NE **Time point:** 24	NA	0.014	10.8	144	0.74	115
**5**	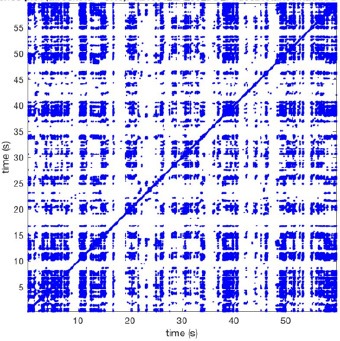	**Wave type:** PW **Origin:** W → E **Time point:** 0 10 39 etc.	NA	NA	0.172	5.17	135	2.19	136
**6**	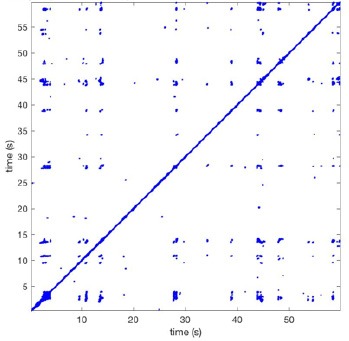	**Wave type:** Collision of 2 PW's **Origin:** N and S **Time point:** 3	NA	NA	0.008	8.20	128	10.3	147
**7**	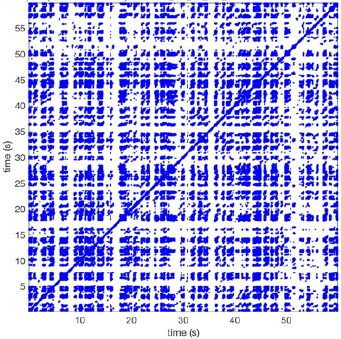	**Wave type:** Collision of 2 PW **Origin:** S and N **Time point:** 5 8 10 etc.	NA	NA	0.200	5.87	127	2.32	151
**8**	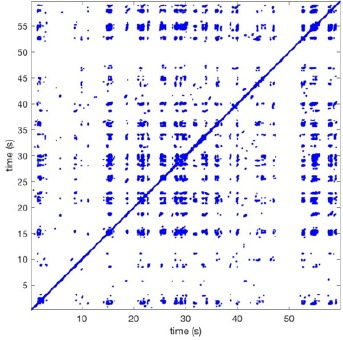	**Wave type:** rBT **Origin:** NE **Time point:** 2 15 25 etc.	**Wave type:** RC **Origin:** Core in the centre **Time point:** 32	NA	0.038	6.03	121	14.0	178
**9**	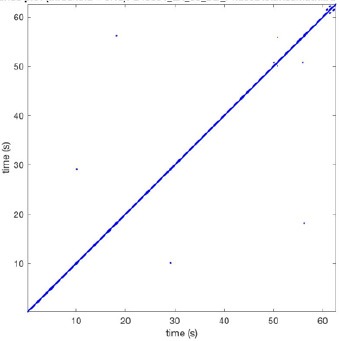	**Wave type:** - **Origin:** - **Time point:** -	NA	NA	0.001	11.2	103	32.4	158
**10**	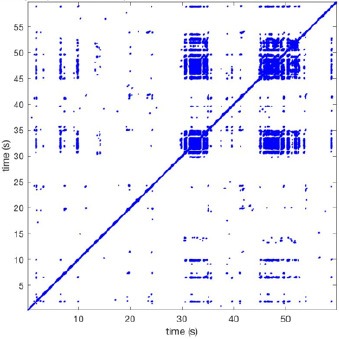	**Wave type:** rBT **Origin:** N **Time point:** 32 48 52	NA	NA	0.039	8.61	139	4.43	144
**11**	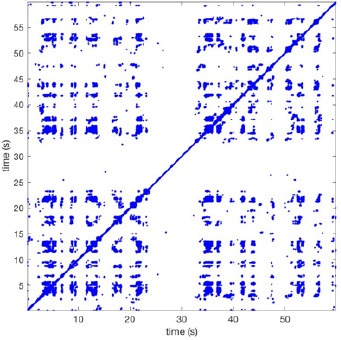	**Wave type:** rBT **Origin:** N **Time point:** 4	**Wave type:** PW **Origin:** NW → SE **Time point:** 21	**Wave type:** Collision of 2 PW **Origin:** South **Time point:** 14	0.049	6.38	122	5.00	129
**12**	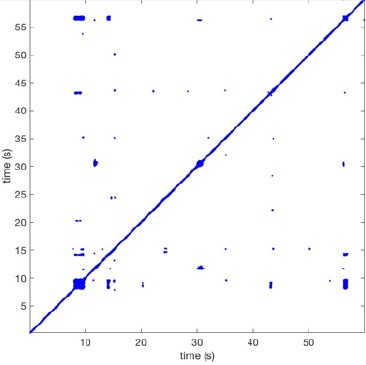	**Wave type:** rBT **Origin:** S **Time point:** 9 56	**Wave type:** rBT **Origin:** N **Time point:** 30	NA	0.006	8.44	102	28.0	157

*Recurrent patterns were subjectively scored to be most frequent (primary), second (secondary) or third (tertiary) in line. For each pattern the wave type, origin and time point of appearance (in seconds) is indicated. Acronyms used are: PW, peripheral wave; rBT, repetitive breakthrough; RC, reentrant circuit; N, north; E, east; S, south; W, west; NA, Not available*.

### AF property maps and effect of time and AF episode

**Figure 8:**
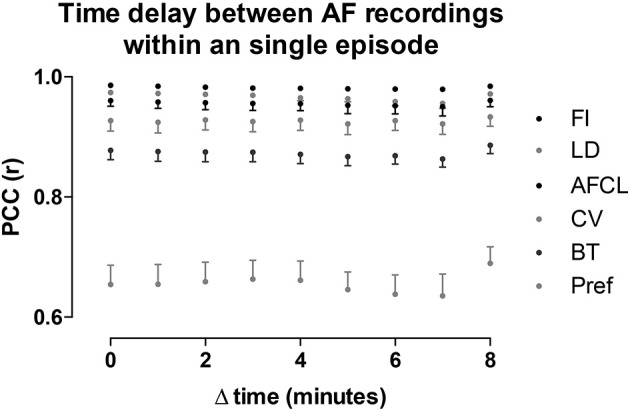
Intra-episode spatiotemporal stationarity of AF parameters. PCC was assessed for all the possible time differences between the 60-s-recordings in a 10-min window. Note, with increasing time difference the number of comparisons decrease, from 9 comparisons at Δt = 1 to 1 comparison at Δt = 9. The parameters are ordered corresponding to the legend order. Data is presented as mean ± sd of the 12 animals.

Potentially, AF property maps could gradually change over a period of several minutes. Therefore, we investigated the effect of time on these property maps. In the section above we determined the average PCC for all comparisons within the 10-min period. Here we averaged the PCC for the different possible time intervals (1–9 min) between the recordings within the 10-min period (Figure 8). No changes in PCC occurred for all parameters at the various time intervals. Thus, within a single AF episode a high degree of spatiotemporal stationarity was found.

**Figure 9:**
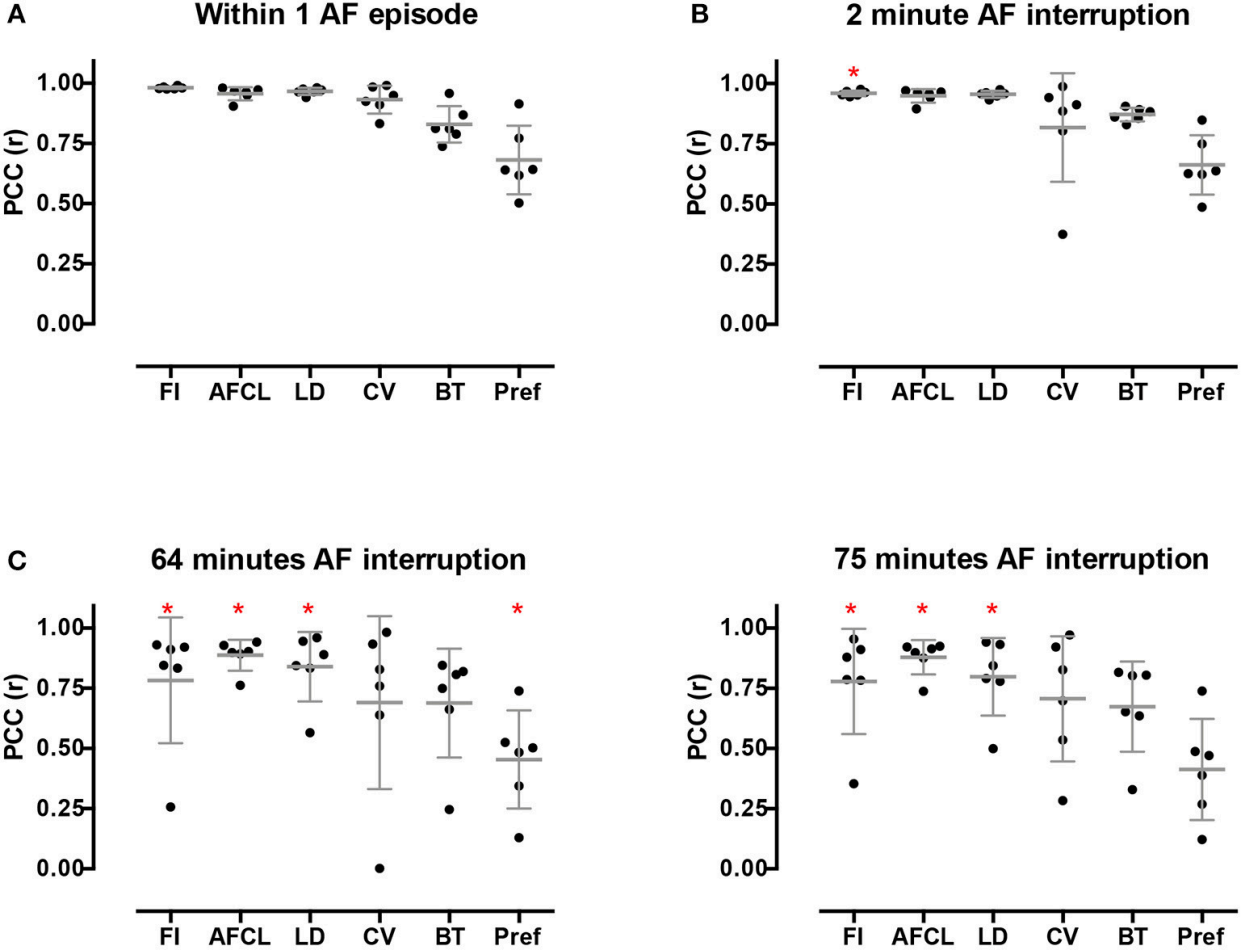
Inter-episode spatiotemporal stationarity of AF parameters. Three different AF episodes were compared. **(A)** Intra-episode stationarity. The average PCC based on the 6 goats in the first episode. **(B)** PCC for inter-episodes stationarity with short time difference. **(C)** PCC for inter-episodes stationarity with ~1 h time difference. Changes in PCC were tested with a Wilcoxon rank sum test without *post-hoc* analysis. ^*^p < 0.05 vs within episode.

High stationarity could reflect the underlying anatomical structure. In that case one would expect that different AF episodes would display comparable AF property distribution. We therefore explored the spatiotemporal stationarity in 2 additional AF episodes (*n* = 6). We chose 2 episodes to be closely apart, 2.1 ± 1 min of AF interruption, and the third episode after ~1h of AF interruption. This resulted into a time difference of 63.4 ± 16.7 min for episode 2 vs. 3 and 75.3 ± 17.9 min for episode 1 vs. 3, (Figure 1, goats 1-6). The 2-min AF interruption led hardly to any changes in average PCC (Figure 9). However, larger changes for some animals occurred than for others, specifically for CV. Interestingly, after the 60-min interruption of AF the PCC led to a large inter-individual variation. The decrease of PCC was significant for AFCL, CV, and FI.

## Discussion

### Major findings

In the goat model of persistent AF, we demonstrated that in 60-s-recordings the spatiotemporal distributions of AF parameters are stationary if measured within a single AF episode. A short interruption of an AF episode had little effect on this stationary behavior but interruption of longer duration changed AF parameters significantly. Sixty-second recurrence analysis illustrated that recurrent patterns were scarce and only found in a minority of the animals. AF was also shown to have highly dynamic behavior at a beat-to-beat level. Therefore, stationary spatiotemporal properties could not be attributed to stable recurrent conduction patterns during AF suggesting that the atrial myocardium may determine average AF properties even when the conduction patterns are highly variable.

### Stationarity of ablation targets

Complex fractionated electrograms (CFAE) were reported to occur during induced AF at sites of conduction block in Wolf-Parkinson-White patients and therefore reflect areas with higher complexity of AF (Konings et al., 1997). Consequently, they might reflect zones that act as sources for AF. It was hypothesized that CFAE sites might be suitable targets for AF ablation (Nademanee et al., 2004). Initially high success rates were reported but recent randomized controlled trials failed to show superiority of PV isolation + CFAE ablation above PV isolation alone (Verma et al., 2015; Vogler et al., 2015). In other studies, high dominant frequency (DF) was found to associate with rotor sites in cholinergic AF in sheep hearts (Berenfeld et al., 2000; Mandapati et al., 2000). This notion led to hypothesis that ablating DF sites might be a valid target for AF ablation (Mandapati et al., 2000; Sanders et al., 2005). Indeed, the presence of high frequency sites inversely correlated to procedural success rates (Gadenz et al., 2017; Kimata et al., 2018) but targeting DF sites did not consistently lead to improvement of clinical outcome (Atienza et al., 2014; Gadenz et al., 2017). It is unclear whether these negative outcomes entail that CFAE and high DF indicate bystander sites instead of AF driver sites or are due to technical limitations of the methods applied.

Irrespective of the applied methods, the intrinsic assumption entail that driver sites are stable in time and space. In most studies, DF and CFAE was determined on electrograms of 2-6s.

Lau et al. (2012), Gadenz et al. (2017) The question is whether these recording lengths are sufficient to capture potential transient appearances of CFAE's or DF's. Salinet et al. reported limited reproducibility of DF zones in non-contact intra-atrial electrograms (Salinet et al., 2014). However, continuous recordings of bipolar contact electrograms showed that about 75% of CFAE's remain stable in time (Lau et al., 2015). Similarly, we found very high spatiotemporal stationarity for both FI and AFCL. Recordings of 60 s within a single episode of AF were more than sufficient to reliably capture patterns. However, the determination of the minimal recording length to obtain representative information was not addressed in this study and would require further investigations, preferably in human recordings.

### Stationarity of conduction properties

Both AFCL and FI are parameters that are derived from single electrograms. Therefore, they reflect AF properties of a limited area. Parameters that account for spatial characteristics of conduction, such as CV and LD had comparably high stationary properties too. Stationarities were lower when AF patterns were taken into account. Preferentiality of conduction direction was subject to largest variability, indicative of dynamic conduction patterns. Breakthroughs were found at almost all electrodes and clear preferential regions of epicardial focal spread of activation were present. Recurrence analysis revealed that half of the goats exhibit periods of repetitive BT's of variable durations. Whether these sites are important for the perpetuation of AF, as proposed by Lee et al. (Lee et al., 2015), warrants further investigation.

In general, AF wave and recurrence analysis revealed multiple propagating wave fronts with a small number of recurrent patterns. Hence, it is unlikely that the high degree of stationary of AF property maps is caused by stable and frequent conduction patterns. Therefore, we conclude that the atrial structure and local electrophysiological properties are likely to determine average AF properties even if the conduction patterns as such are very variable. This might imply that the relatively large variation in AF complexity (3.8–11 waves/cycle), among animals without substantial structural remodeling (Verheule et al., 2010), may—at least partly—be driven by the individual atrial architecture.

### Are AF properties independent from the AF episode?

If AF properties would only be determined by the atrial architecture, then the initiation conditions should not affect AF properties. We therefore examined the effect of different episodes of AF. We found that stationarity is conserved if two AF episodes are relative close in time (~2 min). This observation is in line with Redfearn et al. who found that ~80% of CFAE regions were confirmed when the atria were remapped after a 10 min interval of sinus rhythm (Redfearn et al., 2009). This implies that atrial structure has a prominent role on conduction properties during AF. However, stationarity of AF properties was lower when AF was interrupted >1 h. Because atrial structure does not change in this time interval, we propose that some form of acute recovery from electrical remodeling affected the atrial substrate. This observation might be of clinical importance since some symptomatic AF patients are cardioverted to sinus rhythm before AF ablation. Re-induction of AF during the procedure may reveal other AF patterns or drivers than originally were present with the risk that the leading mechanism cannot be identified.

### The use of recurrence quantification analysis

Recurrence quantification analysis is a technique to identify recurrent states in non-linear dynamical systems. Recurrence quantification analysis has been used in various biomedical disciplines, e.g., postural fluctuation and heart rate variability (Riley et al., 1999; Arcentales et al., 2011). In AF, recurrence analysis has been used mostly for (fractionated) electrogram morphology (Censi et al., 2000; Ng et al., 2014). We made a first step toward recurrent conduction pattern analysis by applying principal component analysis on all electrograms from a high-density array (Zeemering et al., 2015). This approach showed a good correlation with the number of waves but lacked directional and wave front information. Hence, recurrence of the degree of complexity but no recurrence of patterns could be discriminated. Here, we introduce a method accounting for both directionality and complexity of conduction patterns. An important finding is that recurrent patterns were scarce in the overall data set. The frequency of recurrent patterns was inversely related to the number of waves. Recurrent patterns mainly occurred in animals with a low degree of AF complexity. A recurrent pattern in animals with a higher average number of waves per cycle is more likely to be disrupted by other waves. In addition, if more independent wave fronts are present, than the likelihood that all fronts show a recurrence, i.e., synchronize in time and space, is relatively low.

### Future perspectives

The introduced recurrence analysis allows addressing AF on a large time scale and may generate new ideas about AF perpetuation. For instance, the identification of a recurrent period within an episode of AF could indicate maintenance of AF by large circuits, while non-recurrent periods reflect more localized mechanisms. In that case the corresponding dimensions of the recurrence area that matches the local mechanism should be found. Alternatively, recurrent periods could reflect large transient reentrant circuits that act as oscillators of the fibrillation process. These resonators may speed up fibrillation rate with consequent breakdown in distant areas of the atria. Recurrent patterns may also occur before cardioversion of AF when AF complexity becomes low.

## Limitations

An obvious limitation of this study is that the recordings were performed in the goat model of persistent AF in the presence of limited structural remodeling. Additional investigations are required to investigate whether similar patterns occur in patients with AF. Also, the effect of the degree of structural remodeling on recurrent patterns should be addressed in future studies.

## Author contributions

AvH conducted the experiments, analyzed data, scientific interpretation, developed the concept, and wrote the article. SZ developed analysis tools, analyzed data, scientific interpretation, developed of the concept, and contributed to the writing of the article. PP scientific interpretation, developed of the concept, and contributed to the writing of the article. JS developed analysis tools, analyzed data. GG conducted experiments. LH analyzed data. MK conducted experiments. BM contributed to the writing of the article. SV scientific interpretation, developed of the concept, and contributed to the writing of the article. US scientific interpretation, developed of the concept, and contributed to the writing of the article.

### Conflict of interest statement

The authors declare that the research was conducted in the absence of any commercial or financial relationships that could be construed as a potential conflict of interest.
